# Tracking the Biogenesis and Inheritance of Subpellicular Microtubule in *Trypanosoma brucei* with Inducible YFP-**α**-Tubulin

**DOI:** 10.1155/2014/893272

**Published:** 2014-03-30

**Authors:** Omar Sheriff, Li-Fern Lim, Cynthia Y. He

**Affiliations:** ^1^Department of Biological Sciences, National University of Singapore, Singapore 117543; ^2^Centre for BioImaging Sciences, National University of Singapore, Singapore 117546

## Abstract

The microtubule cytoskeleton forms the most prominent structural system in *Trypanosoma brucei*, undergoing extensive modifications during the cell cycle. Visualization of tyrosinated microtubules leads to a semiconservative mode of inheritance, whereas recent studies employing microtubule plus end tracking proteins have hinted at an asymmetric pattern of cytoskeletal inheritance. To further the knowledge of microtubule synthesis and inheritance during *T. brucei* cell cycle, the dynamics of the microtubule cytoskeleton was visualized by inducible YFP-**α**-tubulin expression. During new flagellum/flagellum attachment zone (FAZ) biogenesis and cell growth, YFP-**α**-tubulin was incorporated mainly between the old and new flagellum/FAZ complexes. Cytoskeletal modifications at the posterior end of the cells were observed with EB1, a microtubule plus end binding protein, particularly during mitosis. Additionally, the newly formed microtubules segregated asymmetrically, with the daughter cell inheriting the new flagellum/FAZ complex retaining most of the new microtubules. Together, our results suggest an intimate connection between new microtubule formation and new FAZ assembly, consequently leading to asymmetric microtubule inheritance and cell division.

## 1. Introduction

Trypanosomes are early divergent unicellular protists with a digenic lifecycle successively proliferating in an insect vector and a mammalian host, with several transitional forms. The* Trypanosoma brucei* cell division cycle has been subject to careful investigation. One fascinating feature of* T. brucei* division is the biogenesis and inheritance of a subpellicular microtubule cytoskeleton, which provides the structural basis for the highly organized and polarized* T. brucei* cell body and accurate temporal and spatial duplication of subcellular organelles [1, 2].

The subpellicular microtubule network comprises longitudinal arrays of *α*/*β*-tubulin heterodimers cross-linked to each other as well as to the plasma membrane via various microtubule-associated proteins [3–5]. The microtubule cytoskeleton follows a helical pattern along the long axis of the cell body and once formed remains extremely stable, without apparent disassembly at any time of the cell cycle [6, 7]. The microtubules in the array originate from microtubule organizing centres (MTOC) occupying various niches, each with the potential to be regulated independently [8, 9]. While most of the subpellicular microtubules originate from the anterior region of the cell body and extend posteriorly with their plus ends congregated at the posterior tip of the cell, four specialized microtubules known as the microtubule quartet (MtQ) are nucleated close to the basal body/probasal body complex that also nucleates the flagellum axoneme, extending anteriorly and ending at the anterior tip of the cell [1, 10]. The MtQ is closely associated with an electron-dense filamentous structure, together forming the flagellum attachment zone (FAZ).

During the cell cycle, a new FAZ is assembled together with the new flagellum, posterior to the existing flagellum/FAZ. As the new flagellum/FAZ elongates coordinately, the cell body extends longitudinally, accommodating duplication and segregation of intracellular organelles such as the kinetoplast (condensed mitochondria DNA) and the nucleus. Once the new flagellum/FAZ reaches the same length as the old structures, cell division initiates at the anterior tip of the cell body that is possibly defined by the distal tip of the new FAZ. Cell division then proceeds posteriorly following a helical path between the old and new flagellum/FAZ, and cleaves the cell into two daughters [1, 11, 12]. One daughter inherits the old flagellum/FAZ and the other inherits the newly formed flagellum/FAZ.

The FAZ is tightly linked to subpellicular microtubule biogenesis and organization. Inhibition of FAZ assembly by RNAi depletion of an integral FAZ component CC2D inhibits subpellicular microtubule synthesis, generating a new daughter cell possessing a new flagellum with a shorter cell body [12]. Recent efforts in characterizing the stages of cytokinesis [13] have revealed penetration of microtubules between the new and the old FAZ, in addition to extensive microtubule modifications at the posterior ends of both the daughter cells. However, the construction of the subpellicular microtubule cytoskeleton during cell cycle progression has not been observed directly. Studies on microtubule dynamics have relied heavily on YL1/2, a monoclonal antibody directed against the tyrosinated C-terminal end of *α*-tubulin [14]. Since this tyrosine is subjected to removal by a carboxypeptidase upon incorporation of *α*/*β*-tubulin dimers into the microtubule, YL1/2 has been used as a marker for newly formed microtubules [14]. Based on YL1/2 labelling pattern, a semiconservative model has been proposed for subpellicular microtubule duplication in* T. brucei*. The increase in cell size during duplication is therefore deduced to be brought about by intercalation and posterior extension of new microtubules into the existing subpellicular corset [15]. However, the regulation of tyrosination cycles and its effect on microtubule dynamics is not thoroughly understood [16], and the YL1/2 antibody is known to cross-react with another protein,* Tb*RP2 [17]. A new, improved method to monitor microtubule synthesis directly in* T. brucei* is therefore needed.

In this study, we utilize a tetracycline inducible YFP-*α*-tubulin expression system to follow new microtubule synthesis during the cell cycle of the procyclic (an insect-stage)* T. brucei*. A polyclonal antibody against microtubule “plus” end binding protein, EB1, was also used to monitor microtubule dynamics at the posterior end of the cell. Together, the results suggest that new microtubule synthesis during cell duplication occurs mainly in the region between the old and the new FAZ. At cell division, the more posterior daughter cell inherited more of the newly formed microtubules. Consistent with previous observations, segregation of the duplicated microtubule array was correlated with remodelling at the plus ends.

## 2. Materials and Methods

### 2.1. Cell Lines

YTat1.1 procyclic form* T. brucei rhodesiense* was cultured in Cunningham medium containing 15% heat inactivated fetal bovine serum (BD Biosciences) at 28°C [18]. These were used to create a cell line stably expressing YFP-EB1. Other studies, including inducible YFP-*α*-tubulin expression, endogenous replacements of EB1, and inducible RNA interference, were carried out in procyclic 29.13* T. brucei brucei* cells [19] that were maintained in Cunningham medium containing 15% heat inactivated, tetracycline-free bovine serum (clonetech), 15 *μ*g/ml G418, and 50 *μ*g/ml hygromycin at 28°C. Cell proliferation was measured and growth curve was generated as reported earlier [20].

### 2.2. Plasmids Construction and Transfection

For stable protein expression in* T. brucei*, the full-length coding sequence of* T. brucei* EB1 (Tb09.160.1440) or GCP2 (Tb927.10.9770) was amplified from genomic DNA by PCR and inserted after the C terminus of the Yellow Fluorescence Protein (YFP) reporter cloned in the pXS2 vector to obtain YFP-EB1 and YFP-GCP2 [2, 21]. Ty1-tagged EB1 (Ty1-EB1) was also generated using the pXS2 vector. YFP tagged EB1 was used to replace one endogenous allele and was stably expressed using a modified pCR4Blunt-TOPO vector [22]. To do this, a 500 bp 5′-UTR fragment immediately upstream of EB1 start codon was cloned between PacI and HindIII sites. A 500 bp fragment of EB1 coding sequence immediately downstream of the start codon was cloned into BamHI and NsiI sites. The plasmid was then linearized with PacI and NsiI double digestion before transfection. pLEW100 was used for tetracycline inducible expression of YFP-*α*-tubulin (Tb927.1.2340) [19]. For* T. brucei* GCP2 RNAi, an automated, web-based program was used to search for suitable RNAi target [23] (http://trypanofan.path.cam.ac.uk/software/RNAit.html). A 508 bp fragment specific to the GCP2 coding sequence (nucleotide 1470–1977) was amplified and cloned into the p2T7 vector [24]. For stable transfections, 15 *μ*g of linearized plasmid was transfected into YTat1.1 or 29.13 cells by electroporation (1500 V, 25 *μ*F). Stable, clonal cell lines were generated by serial dilution with medium containing appropriate antibiotics.

### 2.3. Immunofluorescence Assays

*T. brucei* cells were washed and resuspended in phosphate buffered saline (PBS, pH 7.4) and settled on cover slips to allow cells to attach to the glass surface. Cells were then fixed and permeabilized with methanol at −20°C. Alternatively, cells were extracted with droplets of freshly prepared PEM buffer (100 mM PIPES, 1 mM EGTA, 0.1 mM CaCl_2_, 1 mM MgSO_4_, pH6.9) containing 1% Nonidet P-40 for 5 min at room temperature, and then fixed with 4% formaldehyde. The fixed samples were blocked with 3% BSA in PBS and then probed with appropriate antibodies: anti-CC2D [12] or monoclonal L3B2 antibody [25] for FAZ, anti-PAR [26] or anti-PFR1 [27] for the paraflagellar rod along the flagellum, and YL1/2 [14] for tyrosinated *α*-tubulin and the basal bodies (AbCam). The kinetoplast and the nucleus were stained with DAPI (2 *μ*g/ml). Images were acquired using Observer Z1 (Zeiss) equipped with a 63X NA1.4 objective and a CoolSNAP HQ2 CCD camera (Photometrics) and processed with ImageJ and Adobe Photoshop. To image the subpellicular microtubules and the FAZ, serial z-stack images were acquired at 0.5 *μ*m interval throughout the entire cell.

### 2.4. Anti-EB1 Antibody

His-tagged EB1 (His-EB1) was generated by cloning the full-length* T. brucei* EB1 coding sequence inframe into the expression vector pET30a+ (Novagen). His-EB1 recombinant protein was then expressed in BL21* E. coli* and affinity-purified using HIS-Select nickel affinity gel (Sigma). The pooled fractions containing His-EB1 were then exchanged into a gel filtration buffer (25 mM Tris pH7.4, 500 mM NaCl) by running the fractions through a Superdex 200 gel filtration column (GE Healthcare). Purity of the purified His-EB1 was assessed using sodium dodecyl sulphate polyacrylamide gel electrophoresis (SDS-PAGE); most His-EB1 protein was recovered in the soluble fraction (data not shown). Purified His-EB1 protein was used for polyclonal antibody production in rabbits, and the affinity-purified immune serum of one rabbit was used in all subsequent experiments.

### 2.5. Cell Motility Assay

TbGCP2-RNAi cells were diluted using fresh culture medium to approximately 10^5^ cells/ml. 10 *μ*l of diluted cell culture was loaded onto a hemocytometer and visualized using a 20X NA0.4 objective within 30 minutes of removal from the 28°C incubator. Images were captured every ~0.5 second for a total of 60 seconds using a high-speed HSM camera (Zeiss). The movement of individual cells was traced using ImageJ software with MtrackJ plugin [28]. The mean velocity of individual cells was calculated based on the total moving distance in 60 seconds.

## 3. Results

### 3.1. Expression and Incorporation of YFP-*α*-Tubulin into Microtubules

In the* T. brucei* genome, the tubulin genes are clustered as 13–18 tandem repeats of identical *α*/*β*-tubulin gene pairs [29, 30] which are highly conserved across the eukaryotes [31]. Epitope tagging of tubulins has been challenging, as GFP fusions at C-terminus of tubulin genes have failed to complement their corresponding null mutants in* Saccharomyces cerevisiae* [32]. Moreover, *β*-tubulin contains a GTP hydrolysis site and its overexpression or the addition of tags has been reported to be lethal in* S. cerevisiae* [33, 34]. Most in vivo studies of the microtubule cytoskeleton have been performed by expressing tagged *α*-tubulin at reduced levels in the presence of endogenous tubulin [32, 35, 36]. Tag locations and expression systems vary depending on the organisms [36]. In* T. brucei*, transient expression of *β*-tubulin with an internal or C-terminal Ty1 tag was successful [37], though stable expression or integration of tagged-*β*-tubulin into the microtubule has not been reported. The C-terminus of* T. bruceiα*-tubulin is subject to the tyrosination cycle [38]; therefore,* T. bruceiα*-tubulin coding sequence was amplified and fused to the C-terminus of a YFP reporter. To monitor new microtubule synthesis and inheritance, recombinant YFP-*α*-tubulin was expressed from a pLew100 vector under the tight regulation of a tetracycline inducible promoter [19]. A similar approach was previously used to study flagellum assembly dynamics in* T. brucei* [39].

**Figure 1:**
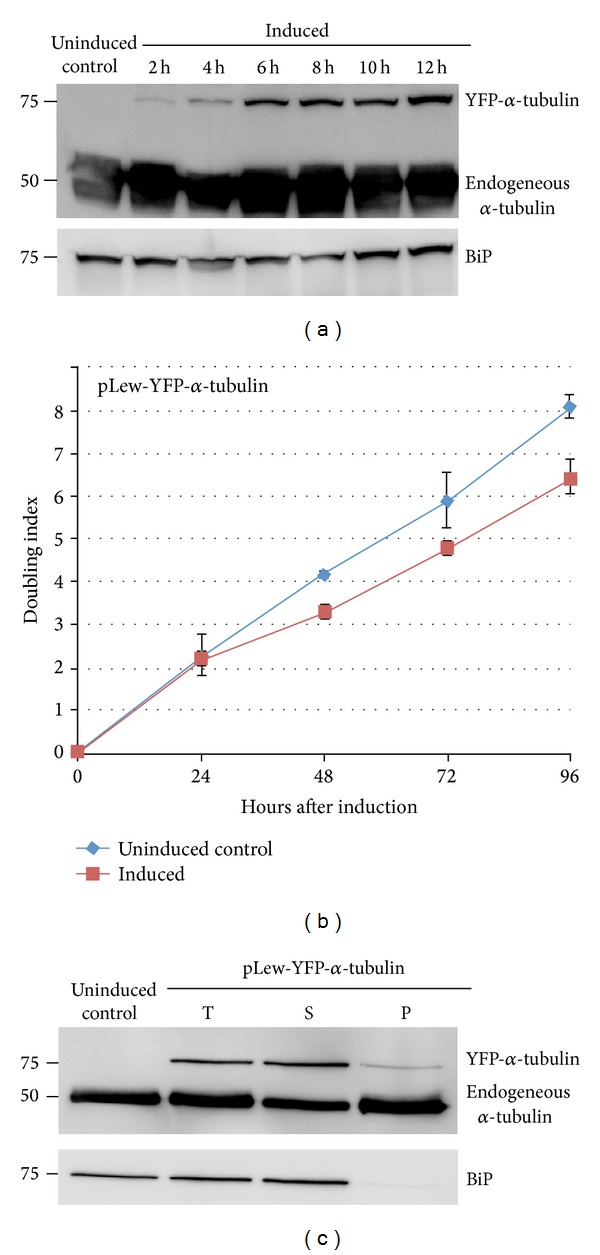
Inducible expression of YFP-*α*-tubulin in* T. brucei*. Cells stably transfected with pLew-YFP-*α*-tubulin were cultivated in the absence or presence of tetracycline to induce YFP-*α*-tubulin expression. Samples were taken at various time points for immunoblots (a), growth curve analyses (b), and cell fractionation studies (c). YFP-*α*-tubulin was detected as early as 2 hours after induction. Continuous induction led to slightly increased YFP-*α*-tubulin level and had little effect on parasite proliferation. Immunoblots of detergent extracted YFP-*α*-tubulin cells indicated that only a small amount of YFP-*α*-tubulin was incorporated into the detergent insoluble cytoskeleton (P). T: total cells; S: detergent soluble fraction.

The inducible expression of YFP-*α*-tubulin protein was monitored by immunoblots with a monoclonal antibody directed against *α*-tubulin (Figure 1(a)). In addition to the endogenous *α*-tubulin at ~50 kDa and a 75 kDa band corresponding to the YFP-*α*-tubulin fusion appeared 2 hours after induction, and the intensity increased over time. Continuous expression of YFP-*α*-tubulin caused no measurable change in doubling time at 24 hours after induction and only a moderate increase in doubling time at later time points (12.3 ± 1.3 hours for uninduced control and 15.0 ± 0.5 hours for induced population) (Figure 1(b)). Induced cells expressing YFP-*α*-tubulin continued to proliferate weeks after induction (data not shown), possibly due to the low expression levels of YFP-*α*-tubulin compared to endogenous *α*-tubulin (Figure 1(a)).

Since the microtubule cytoskeleton of* T. brucei* is resistant to detergent extractions [6], the incorporation of YFP-*α*-tubulin into the microtubule array was verified by immunoblots performed on detergent extracted fractions of control and cells induced for YFP-*α*-tubulin expression for 24 hours (Figure 1(c)). YFP-*α*-tubulin was mostly present in the detergent-soluble fraction and only a small portion was incorporated into the detergent-resistant cytoskeleton fraction. Efficient detergent extraction was verified by the immunolabelling of BiP, an ER luminal protein mostly found in detergent soluble fractions (Figure 1(c)).

### 3.2. Cell Cycle-Dependent Incorporation of New Microtubules

The establishment of a cell line expressing YFP-*α*-tubulin under the control of an inducible promoter provided an ability to monitor the formation of new microtubules in an asynchronous population of cells at various cell cycle stages. Cells induced for YFP-*α*-tubulin expression for 8 hours were extracted with 1% NP-40 in PEM buffer, fixed with 4% formaldehyde, and probed with anti-GFP antibody that cross-reacted with YFP.

**Figure 2:**
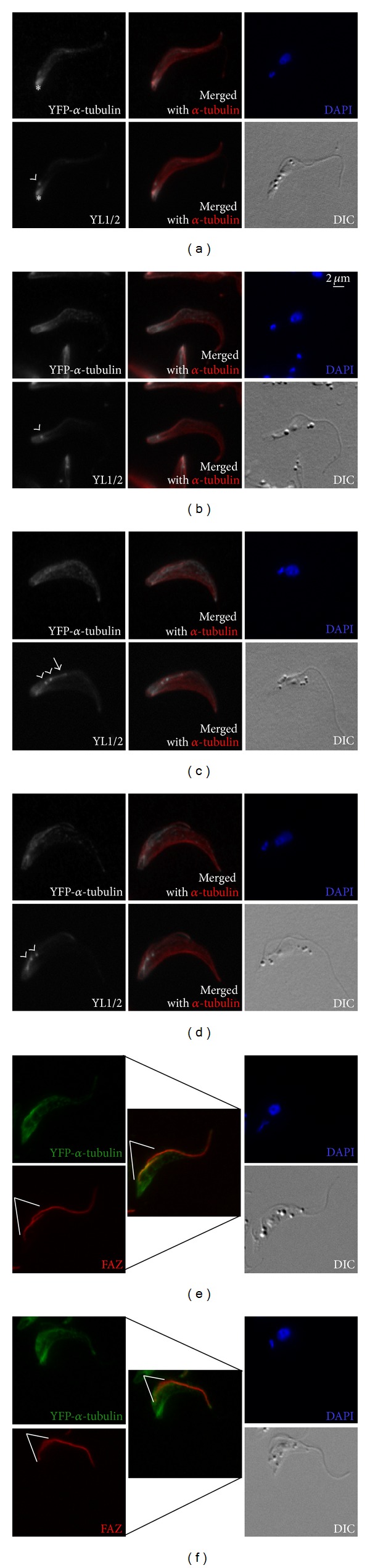
Incorporation of inducible YFP-*α*-tubulin in early cell cycle stages. pLew-YFP-*α*-tubulin cells were induced for 8 hours, extracted with 1% NP-40, and fixed for staining with anti-GFP (for YFP-*α*-tubulin), *α*-tubulin, YL1/2, FAZ and DAPI. In the early cell cycle stage, neither the kinetoplast (small blue dot) nor the nucleus (large blue dot) had duplicated. Basal bodies duplication is one of the earliest events of the cell cycle. * marks the posterior tip of the parasite cell; arrowheads: basal bodies; white lines: new FAZ; arrow: flagellum.

Cells in the early stage of the cell cycle with a single flagellum showed incorporation of YFP-*α*-tubulin at the posterior region of the parasite and weak, speckled labelling in the rest of the cell. This labelling pattern was similar to that of YL1/2, which labeled the basal bodies in addition to the posterior region of the cells (Figures 2(a) and 2(b)). As these cells were likely to be in the late duplication stage at the time YFP-*α*-tubulin was expressed, the YFP-*α*-tubulin and YL1/2 staining patterns suggested low microtubule polymerization activity in late and early cell cycle stages in the cell, except for the posterior region.

As cell cycle progressed, basal bodies duplicated and new flagellum/FAZ emerged. YFP-*α*-tubulin labelling, which was mostly restricted to the posterior region in the earlier stage, now spread toward the anterior part of the cell body (Figures 2(c)–2(f)). Interestingly, YFP-*α*-tubulin staining was more intense on one side of the cell body, along the new FAZ (Figures 2(e) and 2(f)). At this stage, YL1/2 labelling on the duplicated basal bodies and posterior region remained strong. In many cells, YL1/2 also appeared to stain the growing new flagellum and its close proximity as reported previously [13, 40] (Figures 2(c) and 2(d)).

**Figure 3:**
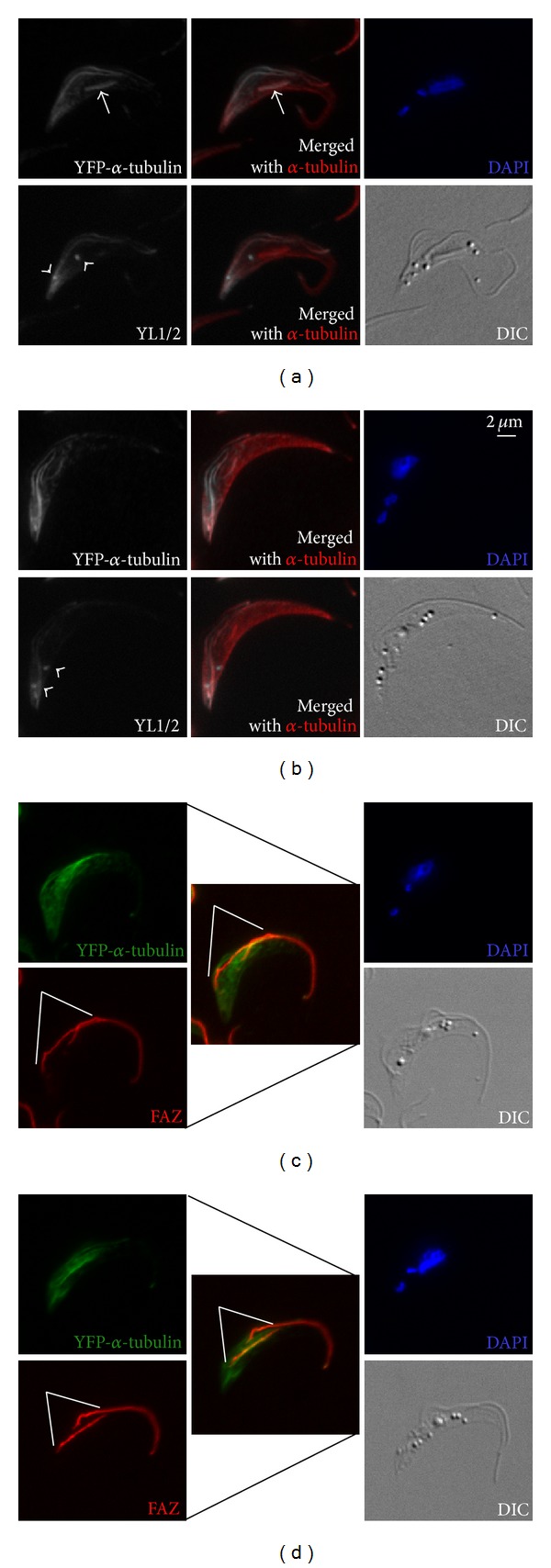
YFP-*α*-tubulin is incorporated primarily in the region between the old and new FAZ in duplicating cells. In the duplicating cells, kinetoplast has duplicated and segregated before the nucleus. Mitotic cells containing an intranuclear spindle can also be observed. Samples were processed as in Figure 2. Arrowheads: basal bodies; white lines: new FAZ; arrow: intranuclear spindle.

As the new flagellum/FAZ complex continued to elongate, basal bodies and associated kinetoplast and flagellum/FAZ segregated (Figure 3). Nuclear division could also be observed in some cells (Figure 3(a)), where YFP-*α*-tubulin was present on the intranuclear mitotic spindle. The increased separation of new and old flagellum/FAZ complexes allowed better visualization of newly synthesized, YFP-*α*-tubulin labeled microtubule in the subpellicular array. Remarkably, strong YFP-*α*-tubulin staining in a striated pattern was found along the new FAZ, particularly in the region between the old and the new FAZ (Figures 3(c) and 3(d)), suggesting active microtubule polymerization in this region. The YL1/2 antibody, similar to the earlier stage, stained the posterior region, the basal bodies, and the elongating new flagellum but not the intranuclear spindle (Figures 3(a) and 3(b)).

**Figure 4:**
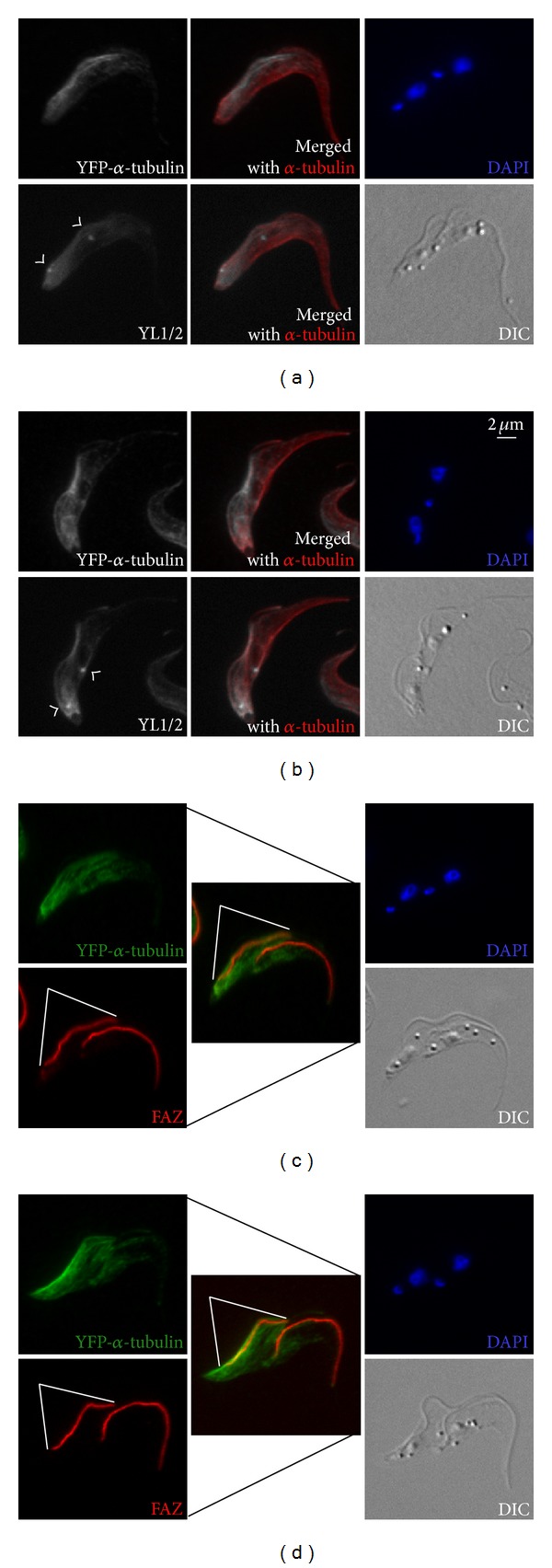
Asymmetric inheritance of newly formed subpellicular microtubules in* T. brucei* cell division. In these postmitotic cells, both kinetoplasts and nuclei have been duplicated and segregated. The partitioning of intracellular organelles and the cytoskeleton network into the daughter cells become evident. Samples were processed as in Figure 2. Arrowheads: basal bodies; white lines: new FAZ.

In cells at later stages of mitosis and those entering cytokinesis, the preferred incorporation of YFP-*α*-tubulin in the region along the new FAZ became even more pronounced (Figure 4). As the formation of the two daughter cells became more evident, the asymmetric segregation of the microtubules also became clear. Whereas the daughter cell inheriting the new flagellum-FAZ complex retained most of the YFP tagged microtubules; the other daughter that inherited the old flagellum/FAZ contained less YFP-labelled microtubules. This asymmetric microtubule biogenesis and inheritance, though could be observed by YL1/2 staining in some cells (Figure 4(b)), was not consistently observed as with YFP-*α*-tubulin [13].

Immunofluorescence of YFP-*α*-tubulin was also performed in cells induced for YFP-*α* tubulin expression for 24 hours (data not shown). In these cells, YFP-*α*-tubulin was found throughout the cell at all cell cycle stages, similar to that of anti-*α*-tubulin antibody. This confirmed the incorporation of YFP-*α*-tubulin into the entire cytoskeleton of* T. brucei* at later time points after induction.

### 3.3. Cellular Localization of* T. brucei* EB1, a Microtubule Plus End Binding Protein

The presence of strong YL1/2 and YFP-*α*-tubulin labelling in the posterior region at all cell cycle stage suggested microtubule dynamics at the plus end, which was then monitored by labelling of EB1, a microtubule plus end tracking protein.* T. brucei* genome encodes a single homologue (Tb09.160.1440) of EB1, which contains an N-terminal calponin homology (CH) domain (amino acids 19–147, with an E-value of 3.5 × 10^−20^), and a C-terminal EB1-like homology (EBH) domain (amino acids 489–534; with an E-value of 3.2 × 10^−14^). Both CH and EBH domains have also been identified in other EB1 proteins [41–43].

**Figure 5:**
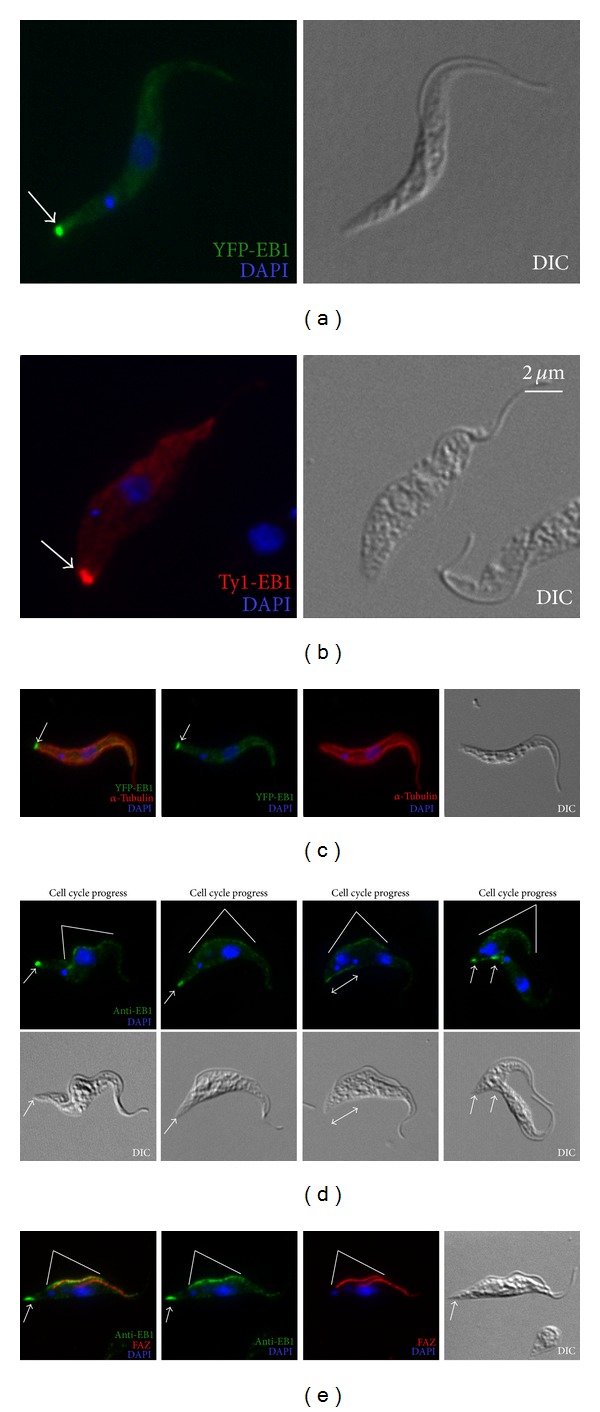
Subpellicular microtubule plus end dynamics revealed by EB1. Cells stably expressing YFP-EB1 (a) or Ty1-EB1 (b) were fixed with cold methanol and labeled with DAPI for DNA. YFP-EB1 cells were also immunolabeled with anti-*α*-tubulin which revealed the total microtubule profile in a parasite cell (c). A polyclonal anti-EB1 was used to label microtubule plus ends throughout the cell cycle (d). Cells double labeled for anti-EB1 and FAZ revealed a possible nonspecific labelling of anti-EB1 along the FAZ region (e). Arrows, EB1 staining at the posterior tip of the cell; double headed arrow: elongated EB1 pattern during mitosis; white lines: possible nonspecific EB1 labelling near FAZ.

In order to establish* T. brucei* EB1's localization within the cell, YFP- or Ty1-tagged EB1 was stably expressed in* T. brucei* cells and produced similar labelling patterns (Figures 5(a) and 5(b)). In most of the cells, specific localization of YFP-EB1 was observed at the posterior tip of the cell, widely accepted to be where the plus ends of the unidirectional corset microtubules converge (Figure 5(c)) [1]. Interestingly, the EB1 labelling pattern varied with cell cycle stages (see Figure S1 in Supplementary Material available online at http://dx.doi.org/10.1155/2014/893272) As new flagellum/FAZ initiated and elongated, YFP-EB1 at the posterior end of the subpellicular array elongated, forming a line that appeared to stretch between the posterior tips of the two daughter cells (Figure S1(d)). The line appeared to lengthen in tandem with the division of nuclei and segregation of the daughter cells. As cell division further progressed, specific YFP-EB1 localization reappeared at the posterior tips of the new daughter cells (Figure S1(e)). It should be noted that a low level YFP-EB1 fluorescence was also observed in the cell body throughout the cell cycle, such a pattern has been previously described for *γ*-tubulin as well [8] (Figure S1). This may represent YFP-EB1 association with subpellicular microtubules other than the plus ends.

### 3.4. Characterization of a Polyclonal Anti-EB1 Antibody

An effect of the GFP tag on the functions of EB1 has been previously reported [44]. To confirm the YFP-EB1 localization, a polyclonal anti-EB1 antibody was raised against purified His-EB1. Affinity-purified anti-EB1 recognized a single band at approximately 57 kDa that corresponded to the expected size of* T. brucei* EB1 in wild type parasite cell lysates. Anti-EB1 also reacted to an additional band at approximately 84 kDa, which corresponded to the expected size of YFP-tagged EB1, in YFP-EB1 cell lysates (Figure S2).

Immunofluorescence staining using the anti-EB1 antibody (Figures 5(d) and 5(e)) revealed a pattern that was mostly consistent with the staining pattern of YFP-EB1. Again, anti-EB1 labeled the posterior tip of the parasite cells during early stages of the cell cycle (Figure 5(d)). As mitosis began and daughter cells formed, the posterior, EB1-containing dot elongated, forming a punctate line joining the posterior ends of the dividing daughters. During cytokinesis, specific anti-EB1 labelling reappeared at the posterior tips of both daughter cells.

Similar to YFP-EB1 labelling, anti-EB1 also showed weak staining in the cell body at all times of the cell cycle. Besides, a weak but consistent staining along the FAZ, particularly the new FAZ, was also observed. The FAZ staining by anti-EB1 was likely nonspecific, as FAZ labelling was rarely observed in YFP-EB1 cells (Figures S1, 5(a) , and 5(c)). Furthermore EB1-RNAi cells that showed reduced anti-EB1 labelling at the posterior tip still retained the FAZ labelling (data not shown).

### 3.5. Role of Microtubule Synthesis on Flagella-FAZ Assembly

By examining the incorporation of inducible YFP-*α*-tubulin into the cytoskeleton, we were able to track new microtubule polymerization and inheritance in* T. brucei*, particularly in duplicating cells. Asymmetric new microtubule synthesis and inheritance was observed and the more posterior daughter cell (that inherited the new flagellum/FAZ complex) retained more newly synthesized microtubules than the other daughter cell.

To further understand how new microtubule synthesis affects* T. brucei* cell cycle progression, particularly the formation of the more posterior daughter cell, Tb927.10.9770, a putative *γ*-tubulin complex 2 protein (GCP2) based on the presence of the characteristic Grip1/2 motifs, was depleted by inducible RNAi [45]. GCP2, GCP3, and *γ*-tubulin form the *γ*-tubulin small complex (*γ*TuSC), important for microtubule nucleation, plus end catastrophe and minus end shrinkage [46, 47].

**Figure 6:**
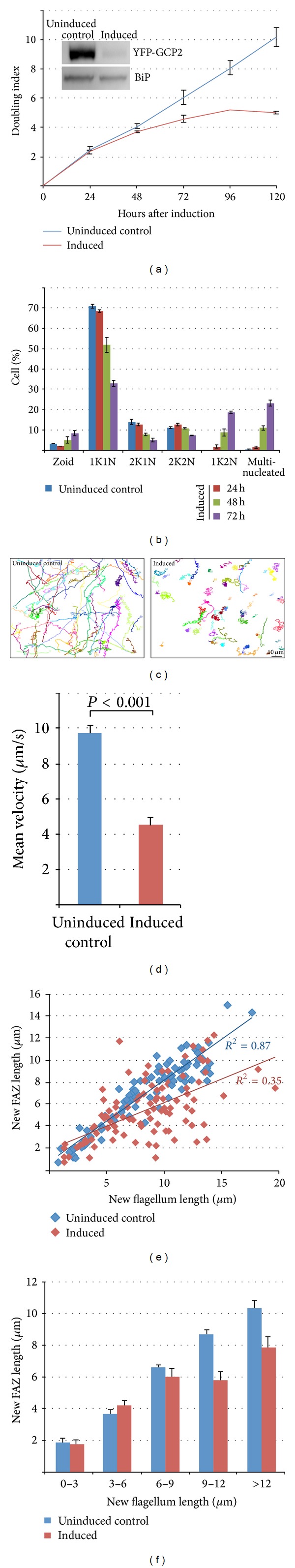
GCP2-RNAi affects new FAZ extension. Cells with a stably integrated GCP2-RNAi construct were grown with tetracycline to induce RNAi or without as control. To monitor the efficiency of RNAi, GCP2-RNAi cells were transfected to allow transient transfection of YFP-GCP2 (a). Samples were then taken every 24 hours after induction for growth assay ((a); results shown as mean ± SD, *n* = 3) and immunoblotting with anti-YFP and anti-BiP (inset). For quantitation of cell cycle effects (b), 400 cells were scored for their DNA contents in each of 3 independent experiments and the results shown as mean ± SD. For motility assays ((c), (d)), uninduced control and cells induced for GCP2-RNAi for 48 hours were diluted in fresh medium, imaged at 2 frames/second for 1 minute, and the movement of individual cells tracked (c) and velocity calculated (d). The 2D-tracks of ~60 cells from three independent experiments were generated by in silico tracking on movies. The velocity results are shown as mean velocity ± SEM of 3 independent experiments with 20–25 cells per experiment. The effect of GCP2 depletion on the new FAZ and flagella elongation was monitored in >100 biflagellated cells in control or cells induced for GCP-RNAi for 48 hours ((e), (f)). The length of new FAZ was plotted against corresponding new flagellum length for each cell measured (e). Alternatively, cells were grouped based on new flagellum length range and FAZ length (shown as mean length ± SEM) was plotted against the flagellum length range (f).

In* T. brucei*, GCP2-RNAi caused a reduction in cell motility and cell proliferation and led to eventual cell death, 96 hours after induction (Figures 6(a) and 6(b)). Microscopic examination of the DNA contents in the GCP2-RNAi population revealed a significant increase of 1K2N cells at 48 hours after induction (*P* < 0.001). At the same time, multinucleated cells also accumulated (*P* < 0.001), suggesting an inhibition of kinetoplast segregation and cell division in GCP2-RNAi cells. Motility tracking indicated a reduction in directional motility and velocity 48 hours after induction (*P* < 0.001) (Figures 6(c) and 6(d)), further supporting an effect of GCP2-RNAi in microtubule-related functions.

In* T. brucei*, both kinetoplast segregation and cell division are microtubule-driven processes that are tightly linked to proper FAZ assembly [1, 12, 48–50] and flagellum motility [51–53]. Cells undergoing new flagellum/FAZ assembly were therefore measured for new flagellum and FAZ length in control and GCP2-RNAi populations. In control cells, new FAZ elongation coordinated with the new flagellum (*R* ^2^ = 0.87), just as previously observed [25]. Upon GCP2-RNAi, this coordinated assembly was disrupted (*R* ^2^ = 0.35 at 48 h after induction) (Figure 6(e)), with the formation of FAZ trailing behind that of the flagellum (Figure 6(f)). This result suggests an effect of new microtubule polymerization on new FAZ assembly.

## 4. Discussion

By inducible expression of YFP-tagged *α*-tubulin,the biogenesis and inheritance of subpellicular microtubules during* T. brucei* cell cycle was monitored. Unlike YL1/2, which reacts to tyrosinated *α*-tubulin and therefore has been used as a probe for microtubule polymerization activities, inducible expression of YFP-*α*-tubulin allowed not only specific labelling of newly synthesized microtubules, but also tracking of their incorporation into the existing cytoskeletal network during cell growth and their inheritance at cell division.

Cellular distribution of inducible YFP-*α*-tubulin indicated that new microtubule incorporation occurred primarily in a region along the new FAZ and between the new and the old FAZs during cell growth. Similar staining was previously reported also for YL1/2, and these observations are consistent with EM observation of new subpellicular microtubules added into the region between the old and the new FAZs [13]. The addition of new microtubules in this region likely mediates the segregation of the basal bodies as wells as other cellular organelles [1, 50] and facilitates the formation of the membrane fold in preparation for cell division [13].

These observations also pointed towards a tight link between new microtubule synthesis and new FAZ formation. FAZ has been previously shown to play a direct role on cell morphology [12]. Cells depleted of an integral FAZ component CC2D could not form a new FAZ. Microtubule polymerization and organization in the region between the old and the new FAZs was also affected and thus generating daughter cells of shorter length [12]. The link between new FAZ assembly and new microtubule formation was further confirmed by GCP2 depletion. It is not clear, however, how new FAZ assembly is coordinated with new microtubule synthesis. One integral component of FAZ is the MtQ, which may be crucial in linking microtubule synthesis to FAZ assembly. One study [8] suggested tight association of *γ*-tubulin along the flagellum, in detergent-resistant manner. Whether *γ*-tubulin or GCP2 may have a structural role in FAZ assembly, as well as function in new microtubule nucleation, remains to be investigated. Additionally, the lagging behind of the FAZ formation with regard to that of the flagellum could be compounded by the motility defect observed in the GCP2 depleted cells. Impaired motility has been identified to be responsible for disruption of basal body migration and its proximal organelles such as the flagellum pocket and the collar [54, 55].

Distribution of YFP-*α*-tubulin in dividing* T. brucei* indicated a distinct, asymmetric inheritance of subpellicular microtubules, with the more posterior daughter cell inheriting most of the newly formed microtubules. This asymmetric inheritance was never observed with YL1/2, possibly due to extensive microtubule plus end remodelling in both daughter cells prior to cell division as previously observed using YFP-XMAP215 as a marker for microtubule plus ends [13]. Using YFP fusion or antibodies to* T. brucei* EB1, a microtubule plus end tracking protein, dynamic microtubule remodeling in the posterior region of both daughter cells was confirmed, particularly during mitosis and cell division stages. The anti-EB1 antibody thus provided a useful tool for monitoring microtubule dynamics in* T. brucei* cells.

## 5. Conclusion

In this current study, we extended the study of subpellicular microtubule biogenesis and inheritance in* T. brucei* by tracking the incorporation of inducible YFP-*α*-tubulin during cell cycle progression. Our results showed that new microtubule synthesis was correlated with new FAZ assembly. Newly formed microtubules were incorporated into the microtubule array primarily in the region between the new and old FAZ. Most of the newly synthesized microtubules were inherited by the more posterior daughter cell that also retained the newly assembled flagellum/FAZ. Polarized new microtubule biogenesis, together with active microtubule plus end remodeling in both daughter cells, led to asymmetric inheritance of subpellicular microtubules in* T. brucei* cell division.

## Supplementary Material


**Figure S1**: **YFP-EB1 localization during *T. brucei* cell cycle**. Cells stably expressing YFP-EB1 (green) were fixed with cold methanol and labeled for FAZ (red) and DNA (blue). Whereas YFP-EB1 was most readily detected at the posterior tip of the cells, YFP-EB1 was occasionally found along the new FAZ. YFP-EB1 labelling at the posterior tip varied with cell cycle progression, forming a dot most of the time (**A, B, C**), elongating into a line at mitosis (**D**), and reforming two separate dots at the posterior tips of the two daughters at cell division (**E**). Arrows, YFP-EB1 staining at the posterior tip of the cell; double headed arrow: elongated YFP-EB1 pattern during mitosis; white lines: occassional YFP-EB1 labelling near the new FAZ.
**Figure S2**: **Characterization of anti-EB1**. Cell lysates from 29.13 control and YFP-EB1 stable cells were fractionated on SDS-PAGE and immunoprobed with anti-EB1 or anti-GFP. Anti-EB1 reacted to a single ~57 KDa band corresponding to the estimated size of *T. brucei* EB1 in wild type 29.13 cell lysates. An additional ~84 KDa band was detected in YFP-EB1 cell lysates and this band was also detected by anti-GFP.
