# Supplementary figures and images for: Tracking the Biogenesis and Inheritance of Subpellicular Microtubule in Trypanosoma brucei with Inducible YFP-α-Tubulin
Source: Biomed Res Int. 2014 Mar 30;2014:893272. doi: 10.1155/2014/893272 (PMC3988969; doi:10.1155/2014/893272)

Cell cycle progress

2  $\mu$ m

DIC

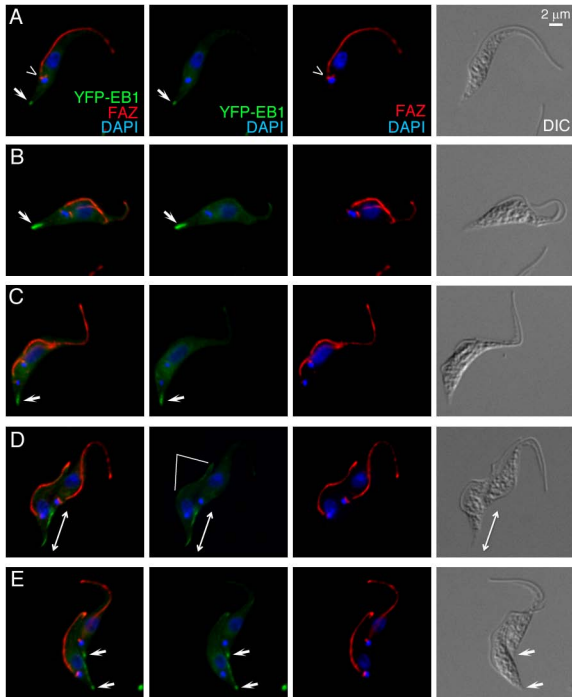

Supplement: Supplementary file 1 — Figure S1: YFP-EB1 localization during T. brucei cell cycle. Cells stably expressing YFP-EB1 (green) were fixed with cold methanol and labeled for FAZ (red) and DNA (blue). Whereas YFP-EB1 was most readily detected at the posterior tip of the cells, YFP-EB1 was occasionally found along the new FAZ. YFP-EB1 labelling at the posterior tip varied with cell cycle progression, forming a dot most of the time (A, B, C), elongating into a line at mitosis (D), and reforming two separate dots at the posterior tips of the two daughters at cell division (E). Arrows, YFP-EB1 staining at the posterior tip of the cell; double headed arrow: elongated YFP-EB1 pattern during mitosis; white lines: occassional YFP-EB1 labelling near the new FAZ. Figure S2: Characterization of anti-EB1. Cell lysates from 29.13 control and YFP-EB1 stable cells were fractionated on SDS-PAGE and immunoprobed with anti-EB1 or anti-GFP. Anti-EB1 reacted to a single ~57 KDa band corresponding to the estimated size of T. brucei EB1 in wild type 29.13 cell lysates. An additional ~84 KDa band was detected in YFP-EB1 cell lysates and this band was also detected by anti-GFP. [file 893272.f1.pdf]

29.13 YFP-EB1  
(control)

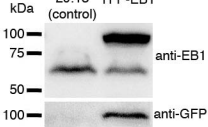

Supplement: Supplementary file 2 [file 893272.f2.pdf]
